# Associations Between Anxiety or Depression Diagnosis and Immune Checkpoint Inhibitor Outcomes

**DOI:** 10.1002/cam4.71326

**Published:** 2025-11-02

**Authors:** Heather Derry‐Vick, Neil J. Shah, Jaeil Ahn, Bianca DeAgresta, Alexandra Della Pia, Jacob P. Zaemes, Lauren Pascual, Natalie Arias‐Orozco, Rachel Zemel, George Sidarous, Michael Serzan, Shaked Lev‐Ari, Adil Alaoui, Alex Marki, Kenna Nguyen, Charalampos Charalampous, Iris Rahman, Olivia Wilkins, Marina Girgis, Aishwarya Sridhar, David Adams, Andrew L. Pecora, Michael B. Atkins, Andrew Ip

**Affiliations:** ^1^ Cancer Prevention Precision Control Institute, Center for Discovery and Innovation Hackensack Meridian Health Nutley New Jersey USA; ^2^ Hackensack Meridian School of Medicine Nutley New Jersey USA; ^3^ Memorial Sloan Kettering Cancer Center New York New York USA; ^4^ Department of Biostatistics, Bioinformatics and Biomathematics Georgetown Lombardi Comprehensive Cancer Center Washington DC USA; ^5^ John Theurer Cancer Center Hackensack University Medical Center Hackensack New Jersey USA; ^6^ Beth Israel Deaconess Medical Center Boston Massachusetts USA; ^7^ MedStar Health Washington DC USA; ^8^ Department of Medical Oncology Dana‐Farber Cancer Institute Boston Massachusetts USA; ^9^ Georgetown Lombardi Comprehensive Cancer Center Washington DC USA; ^10^ Innovation Center for Biomedical Informatics (ICBI) Georgetown University Washington DC USA

**Keywords:** anxiety, cancer, checkpoint inhibitors, depression, efficacy, psychosocial, toxicity

## Abstract

**Introduction:**

Anxiety and depression can affect immune function, yet little is known about their impact on immune checkpoint inhibitor (ICI) therapy outcomes. We investigated associations between an existing anxiety or depression diagnosis and ICI outcomes.

**Methods:**

In this secondary analysis, multicenter retrospective real‐world data were abstracted from medical charts. Patients included received ≥ 1 dose of anti‐PD‐1 or anti‐PD‐L1 monotherapy. Key variables abstracted were anxiety/depression diagnosis at treatment initiation, ICI therapy outcomes (immune‐related adverse events, irAEs; overall survival, OS; time to treatment failure, TTF), and other sociodemographic and clinical factors.

**Results:**

Of the 913 patients, 11% and 12% had an existing anxiety or depression diagnosis, respectively. Rates of any grade irAEs were 32% overall, and 44% and 37% among those with anxiety or depression history, respectively. In the multivariable analysis, patients with an anxiety diagnosis had a greater likelihood of experiencing irAEs (OR = 1.80; 95% CI = 1.16–2.79, *p* = 0.009) and better OS (HR = 0.74; 95% CI = 0.54–1.00, *p* = 0.048) compared to those without an anxiety diagnosis. Depression diagnosis was not significantly associated with irAEs, OS, or TTF. In multivariable sensitivity analyses restricted to patients with non‐small cell lung cancer (NSCLC, *n* = 417), those with an anxiety diagnosis had a trend toward better OS (HR = 0.66; 95% CI = 0.43–1.01; *p* = 0.056) and longer TTF (HR = 0.71; 95% CI = 0.50–1.02; *p* = 0.063) than those without an anxiety diagnosis, while irAEs did not vary significantly by anxiety.

**Conclusion:**

Pre‐existing anxiety diagnosis may impact clinical outcomes for patients receiving anti‐PD‐1 or anti‐PD‐L1 treatments. Links between psychosocial factors and ICI outcomes should be further examined in translational and prospective studies.

## 1 Introduction

Immune checkpoint inhibitors (ICIs) are novel, effective therapies that have changed the cancer treatment landscape across a variety of tumor types. Immune checkpoints regulate immune homeostasis and T‐cell functionality by working against programmed cell death receptor‐1 (PD‐1), PD‐1 ligand (PD‐L1), and other targets [1, 2, 3, 4, 5, 6]. Although ICIs are efficacious, a notable subset of patients experiences immune‐related adverse events (irAEs) or suboptimal tumor responses [7, 8, 9, 10, 11, 12, 13, 14, 15, 16, 17, 18]. It is important to determine which patients experience worse safety and efficacy outcomes on ICI therapy, so that their side effects can be proactively managed, and they can be prioritized for new treatment and/or treatment management approaches.

Existing research focuses primarily on biomarkers or non‐modifiable factors associated with risk for adverse events and poor ICI efficacy outcomes [19, 20, 21, 22]. Psychosocial risk factors such as anxiety and depression can also affect cancer‐related outcomes. In a meta‐analysis of data from over 2.6 million people, those with clinically diagnosed depression or anxiety had a 21% greater risk of cancer‐specific mortality and a 24% greater risk of all‐cause mortality than those without an anxiety or depression diagnosis [23]. Possible behavioral pathways linking depression and anxiety to poorer cancer‐related outcomes include barriers to engaging in healthy behaviors (e.g., physical activity), completing treatment, and coping with stress [24, 25, 26, 27]. Depression and anxiety can also dysregulate immune function and hypothalamic–pituitary–adrenal (HPA) axis function (such as systemic cytokine, cortisol, and catecholamine levels), which could impact irAE development and ICI efficacy [28, 29, 30, 31, 32].

Although anxiety/depression is linked to cancer‐related outcomes and immune function, these patterns have been under‐investigated in the context of ICI therapy. Emerging research suggests that those with emotional distress have worse outcomes during ICI treatment. Among patients with melanoma receiving neoadjuvant ICI therapy in the PRADO trial, those with higher pretreatment emotional distress had fewer major pathologic responses and less 2‐year relapse‐free survival than those with lower emotional distress [33]. Similarly, in a prospective observational study of patients receiving ICI treatment for non‐small cell lung cancer (NSCLC), those with clinically significant anxiety or depression symptoms had shorter median progression‐free survival (PFS), lower objective response rate, shorter 2‐year overall survival (OS), and poorer quality of life than those without depression or anxiety [34]. These key studies establish links between emotional distress and ICI outcomes in a clinical trial and an observational study, which often represent a unique subset of patients receiving cancer care. Examining relationships between depression/anxiety and ICI therapy outcomes in real‐world data is a critical next step.

Taken together, prior studies suggest that psychosocial factors play a role in cancer‐related outcomes and immune dysregulation, which may influence ICI treatment responses. To determine these relationships in the standard treatment setting, we investigated whether an existing anxiety or depression diagnosis influenced ICI safety and efficacy. We examined whether an anxiety or depression diagnosis at treatment initiation was associated with irAEs, OS, and time to treatment failure (TTF) in patients who initiated ICI monotherapy across multiple cancer types. We also conducted sensitivity analyses in patients with NSCLC, the largest subset of patients in this cohort, to examine these questions in a more homogeneous sample with comparable OS and TTF.

## 2 Methods

### 2.1 Study Design and Cohort Selection

This was a secondary analysis of a multicenter, retrospective study of adult cancer patients at 5 MedStar Health hospitals and Hackensack University Medical Center who received at least 1 dose of ICI treatment from January 2011 to April 2018 with follow‐up until January 2021 [6, 15]. This analysis was restricted to patients in the study who received treatment with anti‐PD‐1 or anti‐PD‐(L)1 monotherapy. The Georgetown University Medical Center Institutional Review Board approved the study (IRB 2017–0559) and waived the requirement for informed consent for this non‐interventional study utilizing routinely collected data for secondary research purposes.

### 2.2 Outcome Measures

A comprehensive REDCap (Research Electronic Data Capture) database was developed for data collection, and structured data were captured from the electronic health record using SQL queries [6, 15]. Chart data abstraction included sociodemographic factors, clinical factors (e.g., diagnosis of any anxiety or depression at treatment initiation, number of metastatic sites), occurrence and type of irAEs, date of ICI treatment initiation, and date of death, referral to hospice, or change in systemic therapy, whichever occurred first. A team of data coordinators, research nurses, medical students and resident physicians (overseen and reviewed by physicians from Hackensack University Medical Center and Georgetown Lombardi Comprehensive Cancer Center) used the Common Terminology Criteria for Adverse Events v4.03 to define types and grades of irAEs. OS was defined as the time from the start of ICI treatment to the date of death or censored at the time of last follow‐up. TTF was defined as the time from the start of ICI treatment to death, referral to hospice, or change in systemic therapy, whichever occurred first.

### 2.3 Statistical Analysis

Baseline sociodemographic and clinical characteristics were descriptively summarized. The effects of anxiety or depression diagnoses were examined in separate models due to potential unique effects and the small number of patients with comorbid anxiety and depression diagnoses (*n* = 41, 4%). Univariate analyses (UA) such as Pearson's chi‐squared test or Fisher's exact test for categorical variables were used to examine bivariate relationships between anxiety/depression diagnosis, sociodemographic, and clinical variables with the incidence of any grade irAEs, OS, and TTF; these potential covariates were selected based on prior literature and links demonstrated in the overall cohort study [6]. Given univariate links between anxiety diagnosis and outcomes, multivariable logistic regression analyses followed, which adjusted for age, sex, race, and the variables with *p*‐values < 0.1 in the UA. Depression diagnosis was not examined in multivariable analyses because it was not significantly associated with any of the three outcomes in univariate analyses (p‐values > 0.300). Missing covariates were not imputed in multivariable analyses because > 95% covariate data was available for all multivariable data analyses. For exploratory analyses with OS and TTF time‐to‐event endpoints, multivariable adjusted Cox proportional hazard models were conducted.

Due to anxiety's observed associations with irAEs, OS, and TTF, as well as literature suggesting that irAEs may signal treatment efficacy, we conducted post hoc analyses to examine whether anxiety's association with OS and TTF was mediated by irAEs. To perform mediation analysis for the time‐to‐event data, we followed the work by Scheike et al. [35] and implemented *mets* and *medFlex* R packages. We conducted four mediation analyses: (1) overall sample with OS as the outcome, (2) overall sample with TTF as the outcome, (3) NSCLC subsample with OS as the outcome, and (4) NSCLC subsample with TTF as the outcome, respectively. Please see Figure S1.

A two‐sided test with *p*‐value < 0.05 was used for statistical significance. All analyses were conducted using R software (v.4.3; R Core Team, Vienna, Austria).

## 3 Results

### 3.1 Patient Characteristics

In the overall sample, 913 patients with various cancer types received anti‐PD‐1 or anti‐PD‐(L)1 monotherapy. Patients primarily had lung cancer (46%), melanoma (12%), or gastrointestinal cancer (10%). Patients most commonly received nivolumab (58%), pembrolizumab (35%), or atezolizumab (5%). The median age was 67.4 years (IQR 59.0, 75.7), 57% were male, and 65% were White (Table 1). There were 102 (11%) with a pre‐existing anxiety diagnosis and 106 (12%) patients with a pre‐existing depression diagnosis; 41 patients (4%) had both anxiety and depression diagnoses.

**TABLE 1:** Patient characteristics for overall sample (*N* = 913) and NSCLC subsample (*N* = 417).

Characteristic	Overall sample (*N* = 913), *n* (%)	NSCLC subsample (*N* = 417), *n* (%)	*p*
Age, median years (IQR)	67.4 (59.0, 75.7)	69.9 (61.3, 76.1)	**0.002**
Gender			0.064
Female	394 (43)	204 (49)	
Male	516 (57)	213 (51)	
Race			**0.035**
Black	191 (21)	113 (27)	
White	592 (65)	255 (61)	
Other race	130 (14)	49 (12)	
Anxiety diagnosis	102 (11)	47 (11)	1.000
Depression diagnosis	106 (12)	50 (12)	0.914
Autoimmune disease diagnosis^a^	146 (16)	62 (15)	0.659
BMI ≥ 30 kg/m^2^	174 (19)	68 (17)	0.250
Smoking history^b^	566 (62)	343 (82)	**< 0.0001**
Number of metastatic sites			0.777
0–1	278 (34)	134 (34)	
2	250 (30)	124 (32)	
3 or more	293 (36)	131 (34)	
Pre‐treatment ECOG			0.310
0–1	684 (75)	301 (73)	
≥ 2	224 (25)	114 (27)	
Line of therapy			0.006
1	215 (24)	96 (23)	
2	428 (47)	230 (55)	
3 or more	269 (29)	90 (22)	

*Note:* Bold values indicate *p* < 0.05.

Abbreviations: BMI, Body Mass Index; ECOG, Eastern Cooperative Oncology Group; IQR, interquartile range.

^a^ AID = included a diagnosis of a variety of autoimmune conditions, such as Hashimoto's disease, primary biliary cirrhosis, hypothyroidism, pyoderma gangrenosum, and thyrotoxicosis.

^b^ Smoking history = patients with active or previous/occasional smoking history.

Compared to those without an anxiety diagnosis, patients with an anxiety diagnosis were more likely to be White (*p* = 0.0002) and female (*p* = 0.0002), slightly younger (*p* = 0.059), and slightly more likely to be obese (*p* = 0.062; Table S1). Patients with a depression diagnosis were younger (*p* = 0.005) and more likely to be White (*p* = 0.0006) and female (*p* = 0.021) compared to those without a depression diagnosis. Patients with and without a diagnosis of anxiety or depression did not differ significantly according to their smoking history, autoimmune disease history, number of metastatic sites, lines of therapy, or performance status (*p*‐values > 0.117).

The NSCLC subsample consisted of 417 patients with a median age of 69.9 years (IQR 61.3, 76.1); 51% were male, and 61% were White. There were 47 (11%) patients diagnosed with anxiety and 50 (12%) patients diagnosed with depression. Compared to the overall cohort, patients with NSCLC were older (*p* = 0.002), more likely to be Black (*p* = 0.035), more likely to have a smoking history (*p* < 0.0001), and less likely to have 3 or more lines of therapy (*p* = 0.006).

### 3.2 Toxicity

In the overall sample, the rate of any grade irAEs was 32% across all cancer types. The rates of any grade irAEs were 44% in patients with an anxiety diagnosis and 37% in patients with a depression diagnosis. In the univariate analysis, those with an anxiety diagnosis (*p* = 0.008) or autoimmune disease history (*p* = 0.024) were more likely to experience any grade irAEs. Those who were Black or other races (*p* = 0.011) or had poorer performance status (ECOG ≥ 2; *p* < 0.001) were less likely to experience any grade irAEs (Table S2). Depression diagnosis was not associated with any grade irAEs (*p* = 0.299). In the multivariable analysis, patients with an anxiety diagnosis had a higher risk of developing any grade irAEs compared to those without a pre‐existing anxiety diagnosis (OR 1.80, 95% CI 1.16–2.79, *p* = 0.009; Table 2).

**TABLE 2:** Multivariable adjusted analysis of any grade irAEs in overall sample and NSCLC subsample.

Variables	Overall sample		NSCLC subsample	
	Any grade irAEs, OR (95% CI)	*p*	Any grade irAEs, OR (95% CI)	*p*
Age		0.219		0.165
18‐75 years	*ref*		*ref*	
> 75 years	1.01 (1.00–1.02)		1.01 (0.99–1.04)	
Gender		0.673		0.095
Female	*ref*		*ref*	
Male	0.94 (0.70–1.26)		0.69 (0.44–1.07)	
Race		0.071		0.087
Black	0.68 (0.47–0.99)		0.58 (0.34–0.99)	
White	*ref*		*ref*	
Other race	0.72 (0.47–1.11)		0.63 (0.31–1.29)	
Anxiety		0.009		0.348
No	*ref*		*ref*	
Yes	1.80 (1.16–2.79)		1.38 (0.70–2.71)	
Autoimmune disease		0.063		0.018
No	*ref*		*ref*	
Yes	1.43 (0.98–2.08)		1.99 (1.12–3.53)	
Pre‐treatment ECOG		< 0.001		0.011
0‐1	*ref*		*ref*	
≥ 2	0.46 (0.32–0.66)		0.50 (0.30–0.85)	

*Note:* The analytic n in irAE multivariate analysis was 902 in the overall cohort and 413 for NSCLC subsample, with complete covariate data.

Abbreviations: CI, confidence interval; ECOG, Eastern Cooperative Oncology Group; HR, hazard ratio; irAEs, immune‐related adverse events.

In the NSCLC subsample, the rate of any grade irAEs was 30.5% overall. Rates of any grade irAEs were 38% and 28% in NSCLC patients with an anxiety or depression diagnosis, respectively. Anxiety diagnosis or depression diagnosis was not significantly associated with any grade irAEs in the univariate analysis (*p* = 0.222 and *p* = 0.679 respectively; Table S3). In the multivariate analysis, the relationship between anxiety diagnosis and any grade irAEs was not statistically significant (*p* = 0.348).

### 3.3 Overall Survival

In the overall sample, the median OS was 12.4 months (95% CI 10.9–14.7). In the univariate analysis, poorer performance status (*p* < 0.001), multiple lines of prior therapy (*p* = 0.001), and a higher number of metastatic sites (*p* < 0.001) were associated with worse OS. Those with an autoimmune disease history had better OS than those without an autoimmune disease history (*p* = 0.020; Table S2). Anxiety diagnosis or depression diagnosis was not significantly associated with OS in the univariate analysis (*p* = 0.097 and *p* = 0.876 respectively). In the multivariable analysis, patients with an anxiety diagnosis had better OS relative to those without a pre‐existing diagnosis (HR 0.74, 95% CI 0.54–1.00, *p* = 0.048; Table 3).

**TABLE 3:** Multivariable adjusted analysis of OS in overall sample and NSCLC subsample.

Covariates	Overall sample		NSCLC subsample	
	OS, HR (95% CI)	*p*	OS, HR (95% CI)	*p*
Age		0.169		0.407
18‐75 years	*ref*		*ref*	
> 75 years	1.00 (1.00–1.01)		0.99 (0.98–1.01)	
Gender		0.212		0.53
Female	*ref*		*ref*	
Male	1.12 (0.94–1.35)		1.09 (0.84–1.41)	
Race		0.828		0.058
Black	1.06 (0.85–1.33)		0.69 (0.50–0.94)	
White	*ref*		*ref*	
Other race	1.06 (0.80–1.39)		0.96 (0.62–1.48)	
Anxiety		**0.048**		0.056
No	*ref*		*ref*	
Yes	**0.74 (0.54–1.00)**		0.66 (0.43–1.01)	
Autoimmune disease		0.121		0.281
No	*ref*		*ref*	
Yes	0.82 (0.64–1.05)		0.82 (0.56–1.18)	
Number of metastatic sites		**0.001**		**< 0.001**
0‐1	*ref*		*ref*	
2	**1.48 (1.18–1.85)**		**1.79 (1.29–2.47)**	
3 or more	**1.44 (1.16–1.81)**		**1.83 (1.32–2.54**)	
Smoking history	**—**	**—**		0.082
No			*ref*	
Yes			0.75 (0.54–1.04)	
Pre‐treatment ECOG		**< 0.001**		**< 0.001**
0‐1	*ref*		*ref*	
≥ 2	**2.06 (1.69–2.51)**		**1.80 (1.35–2.40)**	
Line of Therapy		**< 0.001**		**0.004**
1	*ref*		*ref*	
2	**1.20 (0.95–1.51)**		**1.43 (1.02–2.00)**	
3 or more	**1.66 (1.28–2.16)**		**1.91 (1.29–2.82)**	

*Note:* The analytic n in OS multivariate analysis was 810 in the overall cohort and 412 for NSCLC subsample, with complete covariate data. Bold values indicate *p* < 0.05.

Abbreviations: CI, confidence interval; ECOG, Eastern Cooperative Oncology Group; HR, hazard ratio; OS, overall survival.

In the NSCLC subsample, the median OS was 6.86 months (95% CI 0.40–60.66). Anxiety diagnosis or depression diagnosis was not significantly associated with OS in the univariate analysis (*p* = 0.320 and *p* = 0.829 respectively; Table S3). In the multivariable analysis, those with an anxiety diagnosis showed a trend toward better OS compared to those without an anxiety diagnosis (HR 0.66, 95% CI 0.43–1.01, *p* = 0.056; Table 3).

### 3.4 Time to Treatment Failure (TTF)

In the overall sample, the median TTF was 4.1 months (95% CI 3.5–4.4). In the univariate analysis, poorer performance status (*p* < 0.001), multiple lines of therapy (*p* < 0.001), and a higher number of metastatic sites (*p* = 0.014) were associated with shorter TTF (Table S3). Anxiety diagnosis or depression diagnosis was not significantly associated with TTF in the univariate analysis (*p* = 0.268 and *p* = 0.982 respectively). In the multivariate analysis, an anxiety diagnosis was not statistically significantly related to TTF (HR 0.89, 95% CI 0.70–1.14, *p* = 0.367; Table 4).

**TABLE 4:** Multivariable adjusted analysis of TTF in overall sample and NSCLC subsample.

Covariates	Overall sample		NSCLC subsample	
	TTF, HR (95% CI)	*p*	TTF, HR (95% CI)	*p*
Age	*ref*	0.287	*ref*	0.690
18–75 years	1.00 (1.00–1.01)		1.00 (0.99–1.01)	
Gender		**0.02**		0.484
Female	***ref***		*ref*	
Male	**1.20 (1.03–1.41)**		1.08 (0.87–1.36)	
Race		0.698		**0.029**
Black	1.07 (0.89–1.30)		**0.73 (0.56–0.95)**	
White	*ref*		***ref***	
Other race	1.07 (0.85–1.35)		**1.** **14 (0.80–1.62)**	
Anxiety		0.367		0.063
No	*ref*		*ref*	
Yes	0.89 (0.70–1.14)		0.71 (0.50–1.02)	
Autoimmune disease		0.262		0.253
No	*ref*		*ref*	
Yes	0.89 (0.72–1.09)		0.83 (0.61–1.14)	
Number of metastatic sites		**0.032**		**< 0.001**
0‐1	*ref*		*ref*	
2	**1.21 (1.00–1.46)**		**1.** **59 (1.20–2.10)**	
3 or more	**1.26 (1.05–1.52)**		**1.71 (1.30–2.25)**	
Smoking history	**—**	**—**	*ref*	0.132
			0.80 (0.61–1.07)	
Pre‐treatment ECOG		**< 0.001**		**< 0.001**
0–1	*ref*		*ref*	
≥ 2	**1.** **85 (1.56–2.18)**		**1.** **64 (1.29–2.09)**	
Line of Therapy		**< 0.001**		**< 0.001**
1	*ref*		*ref*	
2	**1.22 (1.00–1.48)**		**1.** **32 (1.00–1.74)**	
3 or more	**1.68 (1.34–2.10)**		**2.08 (1.49–2.90)**	

*Note:* The analytic n in TTF multivariate analysis was 809 in the overall cohort and 412 for the NSCLC subsample, with complete covariate data. Bold values indicate *p* < 0.05.

Abbreviations: CI, confidence interval; ECOG, Eastern Cooperative Oncology Group; HR, hazard ratio; TTF, time to treatment failure.

In the NSCLC subsample, the median TTF was 3.5 months (95% CI 0.25–58.25). Anxiety diagnosis or depression diagnosis was not significantly associated with TTF in the univariate analysis (*p* = 0.579 and *p* = 0.558 respectively; Table S3). In the multivariate analysis, patients with an anxiety diagnosis had slightly longer TTF compared to those without an anxiety diagnosis, but this pattern was not statistically significant (HR 0.71, 95% CI 0.50–1.02, *p* = 0.063; Table 4).

### 3.5 Exploratory Mediation Analyses

Please see Table S4. In the overall sample, the magnitude of the natural direct effect of anxiety on OS (HR = 0.807, 95% CI: 0.602–1.081) and the natural indirect effect via irAEs (HR = 0.874, 95% CI: 0.848–0.901) was similar, indicative of strong mediation effects. The natural direct effect of anxiety on TTF (HR = 0.945, 95% CI: 0.801–1.115) and the natural indirect effect via irAEs (HR = 0.894, 95% CI: 0.797–1.003) also supported mediation in the overall sample. Similar patterns were observed in the NSCLC subsample, suggesting irAEs mediated the effect of anxiety on OS and TTF in the overall sample and NSCLC subsample.

## 4 Discussion

In this secondary analysis of multicenter retrospective real‐world data, we examined whether an existing anxiety or depression diagnosis was associated with the safety and efficacy of anti‐PD‐1 or anti‐PD‐L1 monotherapy across cancer types. Patients on ICI therapy with an anxiety diagnosis had a greater likelihood of experiencing any grade irAEs, as well as better overall survival, than those without an anxiety diagnosis. This pattern remained after adjusting for sociodemographic and clinical factors. In multivariate sensitivity analyses of the subgroup of patients treated for NSCLC, those with an anxiety diagnosis had a trend toward better overall survival and longer TTF, though these associations were not statistically significant. In contrast, depression diagnosis was not associated with irAEs, OS, or TTF in the overall sample or in the NSCLC subgroup. Accordingly, pre‐existing anxiety diagnosis may impact clinical outcomes for patients receiving ICI therapy. These findings underscore the importance of incorporating psychosocial assessment into routine care [36] and the need for additional studies to further examine links between psychosocial factors and ICI outcomes.

This is the largest study to date (to our knowledge) to examine links between anxiety/depression and ICI outcomes, a significant strength. By including all patients who initiated anti‐PD‐1 or anti‐PD‐L1 monotherapy at multiple centers during the study period, our sample was highly representative and more diverse than prior studies of emotional distress and ICI outcomes. For example, in a prior observational study of emotional distress and ICI outcomes, 92% of the participants were male [34]. In our study, women (43% of the sample), Black patients (21%), those with ECOG performance status ≥ 2 (25%), and those with 3 or more prior lines of therapy (29%) were well represented, a strength since these groups often have limited representation in prospective studies.

Our findings suggest that patients with an anxiety diagnosis may be more likely to experience irAEs during ICI therapy, although this pattern was weaker in the subgroup of NSCLC patients. Consistent with prior findings suggesting that some irAEs often signal an efficacious treatment response [37, 38, 39, 40, 41], we also observed better OS among patients with anxiety. Further, exploratory mediation analyses suggested that the association between anxiety and better OS was at least partially due to higher irAEs. These findings suggest that, in people with an anxiety disorder, anti‐PD‐1 or anti‐PD‐L1 therapy may result in greater CD8 T‐cell activation that promotes tumor responses. While prior research suggests that chronic, long‐term stress impairs immune function and acute, short‐term stress enhances immune responses [42], the literature on the immune effects of anxiety is less developed. Our findings are consistent with emerging work suggesting that anxiety can lead to immune activation. Among healthy young adults, anxiety symptoms were associated with larger innate immune responses to influenza vaccines, as measured by changes in cytokines, CRP, and expression of type 1 interferon and proinflammatory genes [43]. It is also possible that other unmeasured, underlying mechanisms could help explain the observed links between variables (e.g., hormonal pathways). Taken together, our results suggest that people with an anxiety diagnosis may have greater ICI‐induced immune activation at the level of the tumor and systemically than those without anxiety, an area for future studies.

An additional pathway to consider is that pre‐existing anxiety may influence patients' experience of side effects and their ability to report these side effects to their clinical team for proactive management. Individuals with anxiety often have a heightened awareness of their bodies for physical sensations and changes [37, 38, 39, 40, 41]. If patients notice and report side effects more promptly to their clinical team, adverse events (AEs) can be addressed as they arise, rather than after they worsen. This proactive approach helps maintain effective treatment. Although it is unclear whether administration of systemic steroids or other immunosuppressive medications compromise treatment efficacy [15, 44], several studies indicate that managing cancer symptoms proactively is linked to improved outcomes [45, 46] and positively impacts quality of life.

The patterns observed in our study differ from prior literature on anxiety, depression, and ICI outcomes. In patients receiving ICI therapy who were participating in a clinical trial or a prospective observational study, those with emotional distress, depression, and anxiety had worse outcomes [33, 34]. In contrast, we observed improved efficacy of immune checkpoint inhibitors (ICI) among patients diagnosed with anxiety, and depression diagnosis was not related to outcomes. Yet, the patterns we observed between other clinical factors with OS were largely consistent with the broader literature. For example, poorer performance status and greater number of metastatic sites were associated with worse OS, consistent with prior studies [47, 48, 49, 50]. Accordingly, although the direction of the relationship between anxiety diagnosis and OS differed from prior literature, other factors were related to outcomes in the expected pattern.

There are several possible methodological reasons that we observed a different relationship between anxiety and ICI outcomes than prior studies. First, our patient sample was more diverse than prior studies of ICI treatments [33, 34], and greater inclusion of underrepresented groups may reveal a different pattern than previously shown. Second, while prior studies used self‐report measures of current symptoms, our study used anxiety and depression diagnoses obtained via chart review of comorbidity lists. While the chart diagnosis variable has clinical utility, it does not measure current symptoms; a diagnosis could indicate present or past symptoms that were treated or untreated, and some people with significant anxiety symptoms may not have received a diagnosis in their chart. Accordingly, self‐reported anxiety symptoms and chart‐based anxiety diagnosis variables may lead to different findings. Similarly, it is possible that some people with an anxiety diagnosis do not have current symptoms; of note, there is evidence that a prior history of psychological disorders leaves a mark that can continue to impact immune responses and other outcomes [51, 52]. Third, it is possible that patients with anxiety were taking anti‐anxiety medications, such as β‐1 selective beta‐blockers [53, 54], which could help reverse immune dysregulation and enhance ICI effectiveness. Our findings suggest a need for additional prospective, multi‐method studies to better understand how anxiety, depression, and supportive care medications, particularly anti‐anxiety medications, affect ICI therapy outcomes.

While our study's retrospective design offered strengths including real‐world data and a highly representative sample, it also resulted in limitations. By using chart data from the comorbidity lists, we were not able to distinguish the timing of patients' anxiety or depression diagnosis, the method of diagnosis, the extent to which symptoms were controlled or treated, or current symptom levels. Although this limits the precision of the variable, this is balanced by its relevance to real‐world clinical care. In addition, anti‐anxiety medication use and psychological therapy services were not examined in this secondary analysis of available cohort study data, a significant limitation. Our conclusions may be limited by the lack of data on psychiatric treatment, which may have had an impact on immunotherapy outcomes. For example, initial studies suggest that some SSRIs may promote irAEs [55] and have survival benefits among those receiving immunotherapy [56, 57], while benzodiazepines may negatively impact outcomes [58, 59]. There is a need to jointly consider mental health diagnoses alongside relevant treatments, including study designs that collect detailed data on their timing, duration, and indication. Future prospective studies are needed to determine whether these effects are driven by having a history of anxiety, anti‐anxiety medication, and/or its impact on immune dysregulation.

## 5 Conclusion

This study is among the first to show a link between anxiety and ICI therapy outcomes. Among patients with various cancer types who received anti‐PD‐1 or anti‐PD‐(L)1 monotherapy in this retrospective study, patients with an anxiety diagnosis had a greater likelihood of irAEs and better overall survival. Additional prospective studies with comprehensive psychiatric diagnosis and treatment data are needed to further determine how psychosocial factors and supportive care medications impact clinical outcomes for patients receiving ICI therapy, which could ultimately contribute to toxicity management and treatment optimization.

## Author Contributions

**Heather Derry‐Vick:** conceptualization (equal), writing – original draft (equal). **Neil J. Shah:** conceptualization (equal), data curation (equal), investigation (equal), methodology (equal), supervision (equal), writing – review and editing (equal). **Jaeil Ahn:** data curation (equal), formal analysis (lead), investigation (equal), methodology (equal), writing – original draft (supporting). **Bianca DeAgresta:** project administration (equal), writing – original draft (equal). **Alexandra Della Pia:** project administration (equal), writing – original draft (equal). **Jacob P. Zaemes:** data curation (equal), investigation (equal), writing – review and editing (equal). **Lauren Pascual:** data curation (equal), investigation (equal), project administration (equal), writing – review and editing (equal). **Natalie Arias‐Orozco:** data curation (equal), investigation (equal), project administration (equal), writing – review and editing (equal). **Rachel Zemel:** data curation (equal), investigation (equal), writing – review and editing (equal). **George Sidarous:** data curation (equal), investigation (equal), writing – review and editing (equal). **Michael Serzan:** data curation (equal), investigation (equal), writing – review and editing (equal). **Shaked Lev‐Ari:** data curation (equal), investigation (equal), writing – review and editing (equal). **Adil Alaoui:** data curation (equal), investigation (equal), methodology (equal), project administration (equal), supervision (equal), writing – review and editing (equal). **Alex Marki:** data curation (equal), investigation (equal), writing – review and editing (equal). **Kenna Nguyen:** data curation (equal), investigation (equal), writing – review and editing (equal). **Charalampos Charalampous:** data curation (equal), investigation (equal), writing – review and editing (equal). **Iris Rahman:** data curation (equal), investigation (equal), writing – review and editing (equal). **Olivia Wilkins:** data curation (equal), investigation (equal), writing – review and editing (equal). **Marina Girgis:** data curation (equal), writing – original draft (supporting). **Aishwarya Sridhar:** data curation (equal), writing – review and editing (equal). **David Adams:** writing – review and editing (equal). **Andrew L. Pecora:** funding acquisition (equal), writing – review and editing (equal). **Michael B. Atkins:** conceptualization (equal), methodology (equal), supervision (equal), writing – review and editing (lead). **Andrew Ip:** conceptualization (equal), methodology (equal), supervision (equal), writing – review and editing (lead).

## Ethics Statement

The Georgetown University Medical Center Institutional Review Board approved the study (IRB 2017‐0559). The IRB waived the requirement for informed consent for this non‐interventional study utilizing routinely collected data for secondary research purposes.

## Conflicts of Interest

Dr. Atkins declares Advisory Board/Consultant services to: 23ANDME, AbbVie, Agenus, Atreca, Aveo, Beigene, Boehringer‐Ingelheim, Bristol Myers Squibb, Eisai, Exelixis, IO Biotech, Innovent, JAZZ Pharmaceuticals, Merck, Novartis, OncoRena, Pfizer, Pliant Therapeutics, Pyxis Oncology, Roche, SAB Bio, Sanofi, SeaGen, Simcha, Replimune, Syncona, Takeda, and Werewolf Therapeutics and Stock for: Werewolf Therapeutics and Pyxis Oncology. Dr. Zaemes has worked as a consultant for MJH Life Sciences. Dr. Ip declares consultancy, honoraria, and/or speakers' bureau for: MJH Life Sciences, Graticule, AbbVie, Pfizer/Seagen, Kite/Gilead, Curio Life Sciences, and AstraZeneca, and advisory board for: Pfizer/Seagen, Kite/Gilead, and stocks for: COTA.

## Supporting information


**Table S1:** Overall sample characteristics by anxiety diagnosis and by depression diagnosis.
**Table S2:** Univariate analysis in overall sample.
**Table S3:** Univariate analysis in NSCLC subsample.
**Figure S1:** Illustration of exploratory mediation models.
**Table S4:** Summary of natural direct effects and natural indirect effects for exploratory mediation analyses testing immune‐related adverse events as a mediator linking anxiety with overall survival and time to treatment failure.

## Data Availability

The data that support the findings of this study are available on request from the corresponding author. The data are not publicly available due to privacy or ethical restrictions.
