# Supplementary material for: Associations Between Anxiety or Depression Diagnosis and Immune Checkpoint Inhibitor Outcomes
Source: Cancer Med. 2025 Nov 2;14(21):e71326. doi: 10.1002/cam4.71326 (PMC12579804; doi:10.1002/cam4.71326)
Supplement: Supplementary file 1 — Table S1: Overall sample characteristics by anxiety diagnosis and by depression diagnosis. Table S2: Univariate analysis in overall sample. Table S3: Univariate analysis in NSCLC subsample. Figure S1: Illustration of exploratory mediation models. Table S4: Summary of natural direct effects and natural indirect effects for exploratory mediation analyses testing immune‐related adverse events as a mediator linking anxiety with overall survival and time to treatment failure. [file CAM4-14-e71326-s001.docx]

**Supplementary Material**

**Table S1. Overall sample characteristics by anxiety diagnosis and by depression diagnosis**

|  | **Overall sample (n=913)**  **by anxiety diagnosis** | | | **Overall sample (n=913)**  **by depression diagnosis** | | |
| --- | --- | --- | --- | --- | --- | --- |
|  | **No anxiety diagnosis**  **(n=811)** | **Anxiety diagnosis (n=102)** | **p-value** | **No depression diagnosis**  **(n=807)** | **Depression diagnosis**  **(n=106)** | **p-value** |
| Age  Median  (IQR) | 67.70  (59.20 to 76.07) | 66.40  (58.24 to 71.74) | 0.0585 | 67.82  (59.20 to 76.30) | 66.46  (56.26 to 70.78) | 0.0053 |
| Gender  Female  Male | 333 (41%)  477 (59%) | 61 (61%)  39 (39%) | 0.0002 | 337 (42%)  468 (58%) | 57 (54%)  48 (46%) | 0.0208 |
|  |  |  |  |  |  |  |
|  |  |  |  |  |  |  |
| Race  White  Black  Other | 507 (63%) 182 (22%)  122 (15%) | 85 (83%)  9 (9%)  8 (8%) | 0.0002 | 507 (63%)  174 (22%)  126 (16%) | 85 (80%)  17 (16%)  4 (4%) | 0.0006 |
| Anxiety  No  Yes | --  -- | --  -- | -- | 746 (92%) 61 (8%) | 65 (61%) 41 (39%) | <0.0001 |
| Depression  No  Yes | 746 (92%)  65 (8%) | 61 (60%)  41 (40%) | <0.0001 | --  -- | --  -- | -- |
| Autoimmune  disease  No  Yes | 687 (85%) 124 (15%) | 80 (78%) 22 (22%) | 0.1369 | 684 (85%)  123 (15%) | 83 (78%)  23 (22%) | 0.1178 |
| BMI  < 30 kg/m^2^  ≥ 30 kg/m^2^ | 652 (82%) 147 (18%) | 74 (73%)  27 (27%) | 0.0622 | 649 (82%) 147 (18%) | 77 (74%)  27 (26%) | 0.0914 |
| Smoking  history  No  Yes | 305 (38%) 502 (62%) | 38 (37%) 64 (63%) | 1.0000 | 308 (38%) 495 (62%) | 35 (33%) 71 (67%) | 0.3376 |
| Number of metastatic sites  0-1  2  3 or more | 249 (34%) 215 (30%)  261 (36%) | 29 (30%) 35 (36%)  32 (33%) | 0.3895 | 243 (34%) 221 (31%)  256 (36%) | 35 (35%) 29 (29%)  37 (37%) | 0.9211 |
| ECOG  pre-treatment  0-1  2 or more | 613 (76%)  193 (24%) | 71 (70%)  31 (30%) | 0.1932 | 611 (76%) 191 (24%) | 73 (69%) 33 (31%) | 0.1279 |
| Lines of therapy  1  2  3 or more | 190 (23%)  376 (46%)  244 (30%) | 25 (25%)  52 (51%)  25 (25%) | 0.4936 | 189 (23%)  376 (46%)  241 (30%) | 26 (25%)  52 (49%)  28 (26%) | 0.7604 |
| **Abbreviations:**  Anx= Anxiety, BMI= Body Mass Index, Dep= Depression, ECOG= Eastern Cooperative Oncology Group, IQR=interquartile range, PD-L1= Programmed death-ligand 1. | | | | | | |

**Table S2. Univariate analysis in overall sample**

|  | **irAE** | | **OS** | | **TTF** | |
| --- | --- | --- | --- | --- | --- | --- |
|  | **OR (95% CI)** | **p value** | **HR (95% CI)** | **p value** | **HR (95% CI)** | **p value** |
| Anxiety  No  Yes | *ref*  1.77 (1.16 - 2.69) | 0.008 | *ref*  0.79 (0.60 - 1.04) | 0.097 | *ref*  0.88 (0.70 - 1.10) | 0.268 |
|  |  |  |  |  |  |  |
|  |  |  |  |  |  |  |
| Depression  No  Yes | *ref*  1.25 (0.82 - 1.91) | 0.299 | *ref*  0.98 (0.75 - 1.28) | 0.876 | *ref*  1.00 (0.80 - 1.24) | 0.982 |
|  |  |  |  |  |  |  |
|  |  |  |  |  |  |  |
| ECOG  pre-treatment  0-1  2 or more | *ref*  0.47 (0.33 - 0.67) | <0.001 | *ref*  2.01 (1.66 - 2.43) | <0.001 | *ref*  1.80 (1.53 - 2.11) | <0.001 |
|  |  |  |  |  |  |  |
|  |  |  |  |  |  |  |
| Race  White  Black  Other | *ref*  0.60 (0.42 - 0.87)  0.69 (0.45 - 1.05) | 0.011 | *ref*  1.17 (0.95 - 1.43)  1.09 (0.85 - 1.40) | 0.337 | *ref*  1.10 (0.92 - 1.31)  1.05 (0.85 - 1.30) | 0.564 |
|  |  |  |  |  |  |  |
|  |  |  |  |  |  |  |
|  |  |  |  |  |  |  |
| Gender  Female  Male | *ref*  0.88 (0.66 - 1.16) | 0.356 | *ref*  1.07 (0.91 - 1.27) | 0.407 | *ref*  1.13 (0.98 - 1.31) | 0.092 |
|  |  |  |  |  |  |  |
|  |  |  |  |  |  |  |
| Smoking  history  No  Yes | *ref*  1.07 (0.8 - 1.42) | 0.662 | *ref*  0.93 (0.78 - 1.10) | 0.398 | *ref*  0.96 (0.83 - 1.11) | 0.578 |
|  |  |  |  |  |  |  |
|  |  |  |  |  |  |  |
| BMI  < 30 kg/m^2^  ≥ 30 kg/m^2^ | *ref*  1.21 (0.86 - 1.72) | 0.278 | *ref*  0.94 (0.76 - 1.16) | 0.571 | *ref*  0.94 (0.79 - 1.13) | 0.535 |
|  |  |  |  |  |  |  |
|  |  |  |  |  |  |  |
| Autoimmune  disease  No  Yes | *ref*  1.52 (1.06 - 2.19) | 0.024 | *ref*  0.75 (0.59 - 0.96) | 0.020 | *ref*  0.82 (0.68 - 1.01) | 0.057 |
|  |  |  |  |  |  |  |
|  |  |  |  |  |  |  |
| Number of metastatic sites  0-1  2  3 or more | *ref*  0.99 (0.69 - 1.42)  0.77 (0.54 - 1.1) | 0.275 | *ref*  1.48 (1.19 - 1.85)  1.47 (1.18 - 1.83) | <0.001 | *ref*  1.21 (1.01 - 1.46)  1.3 (1.08 - 1.55) | 0.014 |
|  |  |  |  |  |  |  |
|  |  |  |  |  |  |  |
|  |  |  |  |  |  |  |
| Age | 1.01 (1.00 - 1.02) | 0.203 | 1.00 (1.00 - 1.01) | 0.71 | 1.00 (0.99 - 1.00) | 0.728 |
| Lines of therapy  1  2  3 or more | *ref*  1.07 (0.76 - 1.52)  0.87 (0.59 - 1.28) | 0.442 | *ref*  1.18 (0.95 - 1.47)  1.53 (1.22 - 1.93) | 0.001 | *ref*  1.15 (0.95 - 1.38)  1.50 (1.23 - 1.83) | <0.001 |
| **Abbreviations:**  Anx= Anxiety, BMI= Body Mass Index, CI= Confidence Interval, Dep= Depression, ECOG= Eastern Cooperative Oncology Group, HR= Hazard Ratio, irAE= immune-related Adverse Event, OR= Odds Ratio, OS= Overall Survival, PD-L1= Programmed death-ligand 1, Ref= Reference, TTF= Time to Treatment Failure. | | | | | | |

**Table S3. Univariate analysis in NSCLC subsample**

|  | **irAE** | | **OS** | | **TTF** | |
| --- | --- | --- | --- | --- | --- | --- |
|  | **OR (95% CI)** | **p value** | **HR (95% CI)** | **p value** | **HR (95% CI)** | **p value** |
| Anxiety  No  Yes | *ref*  1.48 (0.79 - 2.78) | 0.222 | *ref*  0.81 (0.54 - 1.22) | 0.320 | *ref*  0.91 (0.66 - 1.27) | 0.579 |
|  |  |  |  |  |  |  |
|  |  |  |  |  |  |  |
| Depression  No  Yes | *ref*  0.87 (0.45 - 1.68) | 0.679 | *ref*  1.04 (0.71 - 1.54) | 0.829 | *ref*  1.10 (0.8 - 1.51) | 0.558 |
|  |  |  |  |  |  |  |
|  |  |  |  |  |  |  |
| ECOG  pre-treatment  0-1  2 or more | *ref*  0.55 (0.33 - 0.90) | 0.018 | *ref*  1.75 (1.33 - 2.29) | <0.001 | *ref*  1.58 (1.25 - 1.98) | <0.001 |
|  |  |  |  |  |  |  |
|  |  |  |  |  |  |  |
| Race  White  Black  Other | *ref*  0.52 (0.31 - 0.86)  0.59 (0.29 - 1.19) | 0.021 | *ref*  0.85 (0.64 - 1.14)  0.97 (0.65 - 1.46) | 0.538 | *ref*  0.85 (0.67 - 1.09)  1.12 (0.81 - 1.56) | 0.275 |
|  |  |  |  |  |  |  |
|  |  |  |  |  |  |  |
|  |  |  |  |  |  |  |
| Gender  Female  Male | *ref*  0.60 (0.40 - 0.92) | 0.019 | *ref*  1.09 (0.85 - 1.39) | 0.483 | *ref*  1.06 (0.86 - 1.30) | 0.598 |
|  |  |  |  |  |  |  |
|  |  |  |  |  |  |  |
| Smoking  history  No  Yes | *ref*  1.11 (0.64 - 1.93) | 0.719 | *ref*  0.63 (0.46 - 0.84) | 0.002 | *ref*  0.65 (0.50 - 0.85) | 0.002 |
|  |  |  |  |  |  |  |
|  |  |  |  |  |  |  |
| BMI  < 30 kg/m^2^  ≥ 30 kg/m^2^ | *ref*  1.32 (0.76 - 2.30) | 0.317 | *ref*  1.12 (0.82 - 1.54) | 0.475 | *ref*  1.03 (0.78 - 1.36) | 0.815 |
|  |  |  |  |  |  |  |
|  |  |  |  |  |  |  |
| Autoimmune  disease  No  Yes | *ref*  2.12 (1.22 - 3.68) | 0.008 | *ref*  0.73 (0.51 - 1.04) | 0.080 | *ref*  0.72 (0.53 - 0.98) | 0.037 |
|  |  |  |  |  |  |  |
|  |  |  |  |  |  |  |
| Number of metastatic sites  0-1  2  3 or more | *ref*  0.76 (0.45 - 1.28)  0.70 (0.42 - 1.18) | 0.370 | *ref*  1.49 (1.09 - 2.04)  1.56 (1.14 - 2.13) | 0.008 | *ref*  1.30 (1.00 - 1.70)  1.48 (1.13 - 1.92) | 0.012 |
|  |  |  |  |  |  |  |
|  |  |  |  |  |  |  |
|  |  |  |  |  |  |  |
| Age | 1.02 (1.00 - 1.04) | 0.083 | 0.99 (0.98 - 1.00) | 0.213 | 0.99 (0.98 - 1.00) | 0.229 |
| Lines of therapy  1  2  3 or more | *ref*  1.26 (0.75 - 2.13)  0.93 (0.49 - 1.77) | 0.451 | *ref*  1.25 (0.91 - 1.72)  1.69 (1.17 - 2.43) | 0.018 | *ref*  1.12 (0.86 - 1.46)  1.76 (1.29 - 2.40) | 0.001 |
| **Abbreviations:**  Anx= Anxiety, BMI= Body Mass Index, CI= Confidence Interval, Dep= Depression, ECOG= Eastern Cooperative Oncology Group, HR= Hazard Ratio, irAE= immune-related Adverse Event, NSCLC= Non-Small Cell Lung Cancer, OR= Odds Ratio, OS= Overall Survival, PD-L1= Programmed death-ligand 1, Ref= Reference, TTF= Time to Treatment Failure. | | | | | | |

Immune-related

adverse events (M)

a

b

Anxiety diagnosis (X)

Overall survival (Y)

c’

Natural direct effect = *β*_c_ Natural indirect effect (*β*_c’_)= *β*_a_*β*_b_

Immune-related

adverse events (M)

a

b

Anxiety diagnosis (X)

Time to treatment failure (Y)

c’

Natural direct effect = *β*_c_ Natural indirect effect (*β*_c’_)= *β*_a_*β*_b_

**Figure S1. Illustration of exploratory mediation models.** Our mediation analysis for the time-to-event data followed the work by Scheike and colleagues (2014). We used *mets* and *medFlex* R packages to test each of the above pathways in separate models for the overall sample and the NSCLC subsample, while adjusting for age, performance status, number of metastatic sites, and multiple lines of therapy.

*Reference*: Scheike TH, Holst KK, Hjelmborg JB. Estimating heritability for cause specific mortality based on twin studies. Lifetime Data Anal. 2014 Apr;20(2):210-33. doi:

10.1007/s10985-013-9244-x. Epub 2013 Feb 2. PMID: 23378036.

**Table S4.** Summary of natural direct effects and natural indirect effects for exploratory mediation analyses testing immune-related adverse events as a mediator linking anxiety with overall survival and time to treatment failure.

| Key outcome | Natural direct effect of anxiety on outcome  HR | 95% Confidence interval (CI) | Natural indirect effect via irAEs  HR | 95% CI |
| --- | --- | --- | --- | --- |
| Overall sample | | | | |
| OS | 0.807 | 0.602 to 1.081 | 0.874 | 0.874 to 0.878 |
| TTF | 0.945 | 0.801 to 1.115 | 0.894 | 0.797 to 1.003 |
| NSCLC subsample | | | | |
| OS | 0.790 | 0.591 to 1.055 | 0.894 | 0.872 to 0.917 |
| TTF | 0.927 | 0.788 to 1.091 | 0.911 | 0.814 to 1.021 |
